# Dental Practitioners’ Knowledge and Attitude Towards Ultrasonography: A Cross‐Sectional Study at a South African University

**DOI:** 10.1155/ijod/7601760

**Published:** 2026-01-16

**Authors:** Jessica Simpson, Tineke van Zyl, Robert Barrie, Faheema Kimmie-Dhansay

**Affiliations:** ^1^ Department of Craniofacial Biology, Pathology, and Radiology, Faculty of Dentistry, University of the Western Cape, Western Cape, 7500, South Africa, uwc.ac.za; ^2^ Department of Community Dentistry, Faculty of Dentistry, University of the Western Cape, Western Cape, 7500, South Africa, uwc.ac.za

**Keywords:** dentists, diagnostic imaging, health promotion, oral health, radiography

## Abstract

**Introduction:**

Ultrasonography (USG) offers a safe, affordable and effective diagnostic tool for various dental applications, yet its use in routine dental practice remains limited, particularly in South Africa. This study, guided by the Knowledge–Attitude–Behaviour (KAB) model, assessed dental practitioners’ knowledge and attitudes towards USG at a South African university. The null hypothesis tested whether attitudes and knowledge differed significantly by the year of graduation or academic department.

**Methods:**

A quantitative cross‐sectional study surveyed 108 dental practitioners at the University of the Western Cape (UWC), Faculty of Dentistry using a custom‐developed, expert‐reviewed online questionnaire. Approval was obtained from the Biomedical Research Ethics Committee (BMREC) and UWC faculty officials for the study. Data were collected via Research Electronic Data capture (REDCap) and analysed with Microsoft Excel and STATA. Participation was voluntary, anonymous and conducted with informed consent.

**Results:**

The questionnaire showed acceptable internal consistency for attitudes (*α* = 0.705) and lower consistency for knowledge (*α* = 0.537), as expected due to varied content. Results indicated a 58.3% response rate with generally positive attitudes towards USG, varying across departments. The Craniofacial Biology, Pathology and Radiology department had the highest attitude score, and Maxillofacial and Oral Surgery showed the highest familiarity with USG, with specific questions yielding statistically significant results. Regarding knowledge, the overall score was 71%. Knowledge levels were high in head and neck swellings and salivary gland disorders, while caries had the lowest score. However, no significant differences were observed across graduation years or departments.

**Conclusion:**

Dental practitioners are not only receptive to learning about USG but also enthusiastic about its potential in patient care. By integrating an USG course into the dentistry curriculum, dental practitioners would be empowered with the skills and knowledge needed to effectively harness the benefits of USG, addressing critical healthcare challenges within South Africa and third‐world countries alike.

## 1. Introduction

Ultrasonography (USG) is a non‐invasive imaging modality that uses high‐frequency sound waves to generate real‐time visualisation of soft tissues. Its affordability, portability and lack of ionising radiation make it an appealing diagnostic option in dentistry [1]. Despite these advantages, USG remains largely underutilised in dental practice, especially in resource‐limited environments such as South Africa.

A growing body of evidence demonstrates that USG provides reliable diagnostic information across multiple dental disciplines. In oral medicine and maxillofacial diagnostics, it helps differentiate cystic from solid lesions and distinguish benign from malignant pathology [2]. For cervical lymphadenopathy, USG can accurately identify metastatic involvement by assessing nodal morphology and echogenic characteristics [3]. In endodontics, it offers a useful adjunct in evaluating periapical pathology, particularly when cortical bone has been compromised [4]. USG is also established in the assessment of salivary gland disorders, including obstructive and inflammatory conditions, where it can visualise calculi and soft‐tissue changes [5, 6]. Emerging work suggests that USG may outperform radiographs in detecting interproximal caries, with improvements in both sensitivity and specificity [7]. In implantology, it offers real‐time information on soft‐tissue thickness and anatomical structures relevant to surgical planning [8], and early clinical studies show that ultrasound images can be digitally aligned with intraoral scans to support guided implant placement with accuracy comparable to CBCT‐based workflows [9].

Despite this breadth of potential applications, uptake of USG in dentistry remains slow. A study by Kondrashova et al. [10] reported that, although most dental students valued their USG learning experiences and felt competent operating the device, only a minority anticipated incorporating it into future practice. This illustrates a gap between early exposure and sustained clinical use—likely related to limited curricular emphasis, lack of structured training and uncertainty about USG’s role within traditional dental workflows. Weimer et al. [11] surveyed dental students and found a strong desire for structured, hands‐on ultrasound teaching during undergraduate studies. Students identified core areas they wish to master, including imaging of the teeth, temporomandibular joint (TMJ), salivary glands, assessment of facial fractures, masticatory muscles and floor of the mouth and tongue, which closely mirror essential topics in interdisciplinary diagnostic practice.

This educational deficit is not confined to dentistry alone. Practitioners in medicine, radiography and allied health often receive minimal training in maxillofacial anatomy, limiting their confidence in interpreting dental‐related USG findings. Consequently, imaging referrals may be delayed or misdirected, reducing the diagnostic utility of USG in head‐and‐neck presentations. Brown et al. [12] found that medical students overwhelmingly supported ultrasound‐based anatomy instruction, yet access to equipment and protected training time remain major barriers. When clinicians across disciplines are unfamiliar with the value of USG for oral and maxillofacial conditions, diagnostic pathways become fragmented and opportunities for early detection are lost. Strengthening USG literacy among both dental and medical professionals is therefore essential for cohesive, interdisciplinary diagnostic care.

This issue is particularly relevant in South Africa, where most of the population relies on the public healthcare sector and waiting times for advanced imaging, such as MRI, are substantial [13]. Broader adoption of USG has the potential to ease these diagnostic bottlenecks by enabling accessible, timely and radiation‐free evaluation of many common dental and head‐and‐neck conditions.

Overall, the current evidence points to a clear and growing interest among dental students in receiving structured ultrasound education. As a radiation‐free imaging option capable of assessing soft tissues, identifying inflammatory and cystic changes and evaluating peri‐implant conditions, USG has significant potential to enrich diagnostic accuracy and enhance patient‐centred care. Early integration into dental education may therefore strengthen clinical competence, expand interdisciplinary collaboration and support more precise treatment planning.

Anchored in the Knowledge–Attitude–Behaviour (KAB) model—which suggests that improved knowledge fosters positive attitudes and ultimately shapes clinical behaviour [14]—this study examines dental practitioners’ knowledge and attitudes towards USG. Using a structured questionnaire, it aims to identify knowledge gaps and perceptual barriers influencing current practice and to determine whether these vary by the year of qualification or academic department. The study tests the null hypothesis that there are no statistically significant differences in dental practitioners’ knowledge or attitudes towards USG based on their year of graduation or departmental affiliation.

## 2. Materials and Methods

### 2.1. Study Design and Population

This article followed a quantitative cross‐sectional design, where data were collected from the population at a single point in time without the need for further follow‐up. This approach therefore provided a snapshot of dental practitioners’ knowledge and attitude towards USG at a specific moment.

All part‐time and full‐time dentists working at the Faculty of Dentistry, the University of The Western Cape (UWC), Cape Town, South Africa, were invited to participate in this study.

### 2.2. Sample Size and Procedure

An online questionnaire (Supporting Information 1) was distributed to all dental practitioners at the university (sample size = 108) by the Academic Administration via Human Resources, in accordance with the Protection of Personal Information (POPI) Act (Act 4 of 2013) (permission reference: UWCRP781448). A review of the literature revealed no existing instrument specifically designed to assess dental practitioners’ knowledge and attitudes towards the use of USG in dentistry. Therefore, the questionnaire was developed based on relevant literature on USG applications in medicine and dentistry, along with established principles of educational needs assessment. Items were constructed to reflect key themes identified in previous studies, such as awareness of USG and familiarity with its clinical applications, while also addressing the specific context of dental education and practice in South Africa.

To ensure content validity, the questionnaire was reviewed by a head and neck radiology expert from Stellenbosch University, who assessed the relevance, clarity and comprehensiveness of the items. A pilot test was also conducted with five dental practitioners from various departments within the faculty to evaluate item clarity and ease of completion. Feedback from the pilot informed minor revisions related to wording and layout. The final questionnaire included a combination of Likert‐scale and yes/no items designed to assess both knowledge of USG’s dental applications and attitudes towards its use and related training. As this was an exploratory study, formal reliability testing (e.g., Cronbach’s alpha) was not performed. This limitation is acknowledged and will be addressed in future studies involving a larger validation sample.

Approval for the study was obtained from the Biomedical Research Ethics Committee (BMREC), with permissions from the UWC Dentistry Faculty Dean and Deputy Registrar for questionnaire distribution.

The survey ran from August 7 to September 7, 2023, with multiple email reminders sent to encourage participation.

A power analysis [15–17] was done to obtain a sample size of 108:

Unlimitedn=Zα/2 2p1−pd2=1.962∗0.50.50.052=384.16,

Finiten′=m1+m−1N=384.161+384.161−83=98,

where *n* = sample size, *Z* = statistical level of confidence, *p* = expected proportion and *d* = precision.

If *Z* = 1.96 (95% confidence) and using a prevalence of 50% (1) *P* = 0.5 and *d* = 0.05, *N* = 130 (part‐time and full‐time dental practitioners at the UWC Faculty of Dentistry), the finite *n′* is ~98.

This number is increased by 10% for non‐response. Thus, a sample size of 108 was proposed.

### 2.3. Variables

1. Outcomes (dependent variables)
  • Knowledge score
  • Attitude score
2. Exposures (independent variables)
  • Age group
  • Years of practice
  • Department (Oral and Maxillofacial Surgery, Prosthodontics, etc.)
  • Level of training (undergraduate, postgraduate, specialist)
3. Predictors
  • Prior exposure to USG
  • Availability of USG in the department
  • Previous training in radiology/imaging

### 2.4. Reliability and Validity

Internal consistency of the questionnaire was assessed using Cronbach’s alpha. The attitude section, which measured underlying perceptions related to USG, demonstrated acceptable to good internal consistency (attitude: *α* = 0.705).

The knowledge section yielded a lower Cronbach’s alpha (*α* = 0.537). This result is consistent with the nature of the section, which comprised discrete factual questions spanning a variety of clinical areas (e.g., soft tissue imaging, hard tissue imaging and joint assessment). Given that knowledge of USG in these areas is likely to vary independently among practitioners, high internal consistency was not expected. Cronbach’s alpha is known to be an imperfect measure for factual knowledge tests, where items do not necessarily reflect a single latent concept. Therefore, descriptive statistics (mean scores and percentage correct) were also used to summarise knowledge section results.

### 2.5. Data Collection and Analysis

Data were collected using Research Electronic Data Capture (REDCap). Microsoft Excel was used for data management. This information was then imported into STATA software (StataCorp. 2019. Stata Statistical Software: Release 17. College Station, TX: StataCorp LLC.).

Descriptive statistics (i.e., percentages) and chi‐square tests, or Fisher’s exact test were performed to detect any associations. All tests were deemed statistically significant at *p*  < 0.05.

### 2.6. Ethics Approval and Consent

Ethics approval (reference number BM23/5/1) was granted by the BMREC at the UWC.

Before completing the questionnaire, an information sheet containing all relevant details about this study was included in the introductory part of the email. Once the survey was opened, a consent form, which included the researchers’ and supervisors’ contact information, as well as the POPI Act declaration, was disclosed.

All participation by staff was voluntary. Participants could withdraw from the study at any time and were not penalised for doing so. All information obtained from the questionnaires was anonymous and confidential.

## 3. Results

### 3.1. Participant Characteristics

A total of 63 dental practitioners (response rate = 58.3%) responded and fully completed the survey, all of whom gave consent for their personal information to be collected, stored, processed and shared. Of these 63 individuals, 16 graduated with their dental degree before 2000, while 47 graduated after 2000. The distribution of participants by the department was as follows: the Prosthodontic Department had the highest representation (33.3%), followed by Craniofacial Biology, Pathology and Radiology (19.0%), Maxillofacial and Oral Surgery (17.5%), Orthodontics and Paedodontics (14.3%), Oral Medicine and Periodontology (9.5%) and Community Dentistry (6.4%).

### 3.2. Dental Practitioners’ Attitude Towards USG

#### 3.2.1. Attitude by Year Graduated

Dental practitioners’ attitudes towards USG were compared based on graduation year: ‘before 2000’ and ‘after 2000’. While no significant difference was found (*p*  > 0.05), those graduating before 2000 had a slightly more positive attitude (mean = 3.6 out of 5) towards USG’s utility and potential benefits, such as improving diagnosis and treatment planning and making patient care more efficient.

Those graduating after 2000 had a mean attitude of 3.5 and rated higher in their familiarity with USG as well as their interest in attending accredited USG courses.

#### 3.2.2. Attitude by Department

**Figure 1:**
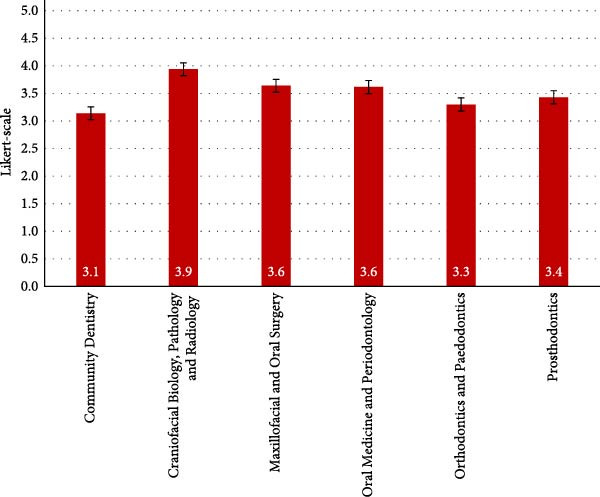
Mean overall attitude scores by department. Mean Likert‐scale ratings (± standard error) of dental practitioners’ overall attitudes towards ultrasonography by department. Scores ranged from 1 (very negative) to 5 (very positive).

Craniofacial Biology, Pathology and Radiology had the highest mean attitude towards USG (3.9), followed by Maxillofacial and Oral Surgery and Oral Medicine and Periodontology (both 3.6), Prosthodontics (3.4), Orthodontics and Paedodontics (3.3) and Community Dentistry (3.1) (Figure 1).

When it came to the department that was most familiar with USG, Maxillofacial and Oral Surgery scored the highest (2,3). Craniofacial Biology, Pathology and Radiology scored highest in recognising USG’s utility in dental diagnosis and treatment planning, openness to its use and interest in accredited courses.

Community Dentistry showed the highest agreement with the necessity of training and USG’s potential cost and time benefits but showed the lowest scores across familiarity and USG’s utility, followed by Orthodontics and Paedodontics for training necessity and USG’s benefits.

Statistical significance was found in attitudes towards USG’s utility (*p* = 0.0185) and its potential improvement in diagnosis and treatment planning (*p*  < 0.001). Significant differences were observed between various departments in these attitudes.

Interest in attending an accredited USG course was statistically significant (*p* = 0.0440), with Craniofacial Biology, Pathology and Radiology showing the highest interest compared to Community Dentistry.

Overall mean attitude was also significant (*p* = 0.0101), with significant differences between Orthodontics and Paedodontics and Craniofacial Biology, Pathology and Radiology, as well as Prosthodontics and Craniofacial Biology, Pathology and Radiology.

#### 3.2.3. Overall Attitude Towards USG

**Figure 2:**
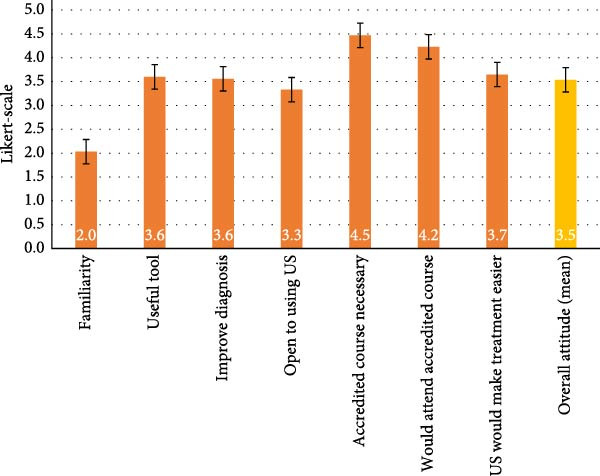
Attitude towards ultrasonography. Mean Likert‐scale ratings (± standard error) of dental practitioners’ attitudes towards various aspects of ultrasonography. Scores ranged from 1 (very negative) to 5 (very positive). Overall attitude is shown as the average across all domains.

The overall attitude towards USG was moderate, with a mean score of 3.5 out of 5 (Figure 2). The question about familiarity with USG had the lowest score at 2.0. The highest scores were for the importance of attending an accredited USG course (4.5) and interest in attending such a course (4.2). Questions about USG’s utility in diagnosis and treatment, openness to its use and potential cost and time benefits scored between 3.3 and 3.7.

### 3.3. Dental Practitioners’ Knowledge Towards the Use of USG

#### 3.3.1. Knowledge by Year Graduated

The ‘before 2000’ age group scored slightly higher in overall knowledge at 75% compared to 68% for the ‘after 2000’ group, with no statistical significance (*p*  > 0.05). The ‘before 2000’ group outperformed in all knowledge areas: periapical lesions (63% vs 57%), head and neck swellings (100% vs 89%), TMJ disorders (94% vs 89%), salivary gland disorders (100% vs 98%), caries (19% vs 15%), cervical lymph nodes (100% vs 96%) and implant placement (50% vs 30%).

#### 3.3.2. Knowledge by Department

All *p*‐values were greater than 0.05, indicating no statistical significance in knowledge among departments.

**Figure 3:**
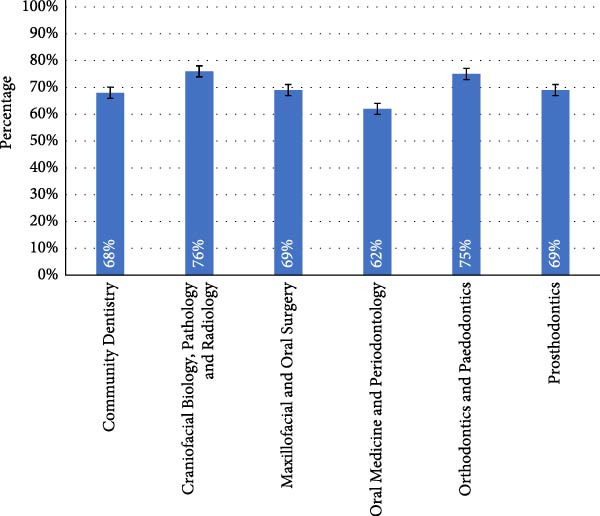
Overall knowledge scores by department. Mean percentages (± standard error) representing dental practitioners’ overall knowledge in each department.

Craniofacial Biology, Pathology and Radiology led in knowledge with 76%, followed closely by Orthodontics and Paedodontics at 75%, and Maxillofacial and Oral Surgery and Prosthodontics at 69%. Community Dentistry scored 68%, and Oral Medicine and Periodontology scored 62% (Figure 3).

Similarly, for TMJ disorders, salivary gland disorders and cervical lymph node assessment, multiple departments scored 100%, with varying scores for Prosthodontics and Oral Medicine and Periodontology. Craniofacial Biology, Pathology and Radiology scored 25% for caries, while Community Dentistry scored the lowest (0%). Orthodontics and Paedodontics scored the highest (56%) for implant placement, while Oral Medicine and Periodontology scored the lowest (17%).

All *p*‐values were above 0.05, indicating no statistical significance in knowledge across departments.

#### 3.3.3. Overall Knowledge of USG

**Figure 4:**
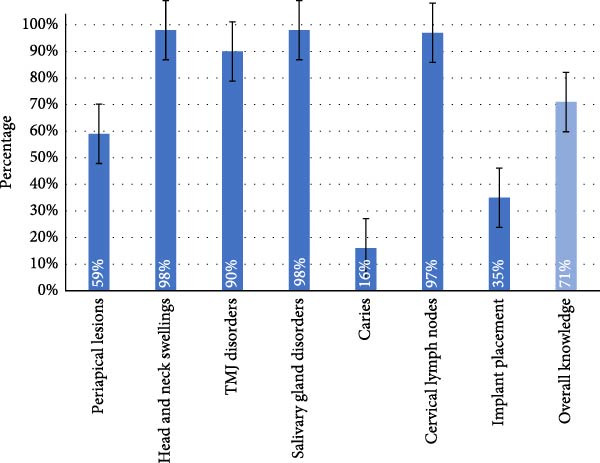
Knowledge of ultrasonography by application. Mean percentages (± standard error) representing dental practitioners’ knowledge of ultrasonography for specific diagnostic and clinical application. Overall knowledge is shown as the average across all domains.

The overall knowledge towards USG was 71% (Figure 4). ‘Head and neck swellings’ and ‘salivary gland disorders’ both scored the highest in knowledge, with a total of 98%. This was followed closely by ‘cervical lymph nodes’ and ‘TMJ disorders’, which scored 97% and 90%, respectively.

‘Periapical lesions’ scored 59% and ‘implant placement’ 35%. ‘Caries’ rated the lowest in overall knowledge, with a combined score of 16%.

## 4. Discussion

A total of 63 out of 130 dental practitioners completed the online questionnaire, producing a response rate of 58.3%, which compares favourably with typical online survey averages of 34% [18] and 44.1% [19]. Most respondents (74.6%) graduated after the year 2000. The Prosthodontics department contributed the highest number of responses, although this likely reflects its larger staff complement within the UWC Faculty of Dentistry rather than differing levels of interest across departments. Conversely, Community Dentistry—being the smallest department—had the lowest participation, which therefore should not be interpreted as reduced willingness to partake in the study.

### 4.1. Dental Practitioners’ Attitude Towards USG

Although differences were not statistically significant, older practitioners showed more interest in incorporating USG into clinical practice but were less inclined to attend accredited training courses. This pattern aligns with broader literature suggesting age‐related challenges in adopting new technologies [20], including cognitive load, slower motor adaptation and reduced confidence with unfamiliar digital tools.

In contrast, younger practitioners expressed greater enthusiasm for attending USG courses, consistent with existing generational trends in digital technology adoption and a generally higher comfort level with acquiring new technical skills.

Departments with greater exposure to diagnostic imaging demonstrated stronger positive attitudes towards USG. The Craniofacial Biology, Pathology and Radiology department—as well as the Maxillofacial and Oral Surgery department—scored highly, likely reflecting their routine reliance on imaging for diagnosis and treatment planning. Community Dentistry reported the lowest attitude scores, which is consistent with its public health‐oriented approach and historically conservative stance towards integrating new technologies [21].

Interestingly, Orthodontics and Paedodontics showed limited interest in USG training despite its radiation‐free nature, which is particularly beneficial for children and special needs populations. This suggests a potential lack of awareness regarding USG’s clinical advantages in vulnerable groups [22].

Overall, the moderate mean attitude score (3.5) likely stems from the absence of undergraduate USG teaching in South African dental curricula [13]. Limited exposure during training can diminish awareness and reduce practitioners’ confidence in adopting emerging imaging modalities. Nonetheless, the strong interest in accredited USG courses mirrors findings by Khodadadi et al. [23], who reported widespread support for ultrasound education among healthcare professionals. This indicates a substantial demand for structured continuing education in dental USG.

### 4.2. Dental Practitioners’ Knowledge Towards USG

Although not statistically significant, older practitioners achieved slightly higher knowledge scores (75%) than younger practitioners (68%). This may reflect accumulated clinical experience, engagement in continuous education and participation in mentoring activities—all of which reinforce long‐term retention of diagnostic concepts.

Respondents demonstrated strong knowledge of USG applications such as head and neck swellings, salivary gland pathology, cervical lymph nodes and TMJ disorders. These areas benefit from interdisciplinary overlap, where ultrasound is well established.

However, knowledge was substantially lower for applications such as periapical lesion assessment, implant planning and caries detection—domains traditionally dominated by radiographic imaging. Limited curricular exposure likely contributes to reduced awareness of USG’s emerging capabilities.

Departments more closely aligned with diagnostic imaging, such as Craniofacial Biology and Maxillofacial and Oral Surgery, demonstrated higher overall knowledge scores. Yet no statistically significant differences were observed across departments, suggesting that although curricular exposure varies in emphasis, baseline diagnostic training remains relatively consistent across the institution.

### 4.3. Limitations

This study focused solely on dental practitioners from UWC Faculty of Dentistry, leading to a smaller sample size compared to all dental universities in South Africa. Distributing the questionnaire via email added complexity, with potential factors like email filtering, practitioner disinterest or technical issues possibly affecting response rates. The anonymous nature of the survey made targeted follow‐ups challenging. Future research could explore better data collection methods, improve questionnaire accessibility and address potential participation barriers to enhance response rates.

## 5. Conclusion

The study highlights ‘a notable lack of dentists’ awareness regarding the benefits of USG in dentistry, revealing an education gap in South Africa. To address this, integrating a dental USG module into undergraduate programs or offering accredited courses for practising dentists is essential. This enhanced knowledge can foster a positive attitude toward USG, leading to improved patient diagnosis and outcomes.

In South Africa, and many third‐world countries alike, this is crucial for addressing the significant disease burden. USG provides solutions to various pressing issues, including long waiting times for CT or MRI scans, high costs, radiation concerns, limited access to imaging tools and logistical challenges. The research underscores dental practitioners’ eagerness to learn and apply USG, supporting the case for integrating it into dental curricula to equip practitioners with vital skills and effectively tackle regional healthcare challenges.

## Disclosure

This research formed part of the requirements for the degree of Master of Science in Dentistry and was completed in September 2024 [24].

## Conflicts of Interest

The authors declare no conflicts of interest.

## Author Contributions

**Jessica Simpson:** conceptualisation, methodology, investigation, writing – original draft, visualisation. **Tineke van Zyl:** writing – review and editing, supervision. **Robert Barrie:** writing – review and editing, supervision. **Faheema Kimmie-Dhansay:** validation, formal analysis, data curation.

## Funding

This research received no specific grant from funding agencies in the public, commercial, or not‐for‐profit sectors.

## Supporting Information

Additional supporting information can be found online in the Supporting Information section.

## Supporting information


**Supporting Information** Online questionnaire sent out to all dental practitioners.  ^∗^Referenced on page 5, under “sample size and procedure”.

## Data Availability

The data that support the findings of this study are openly available in Kikapu at https://doi.org/10.25379/uwc.25751823.v1.
