# Supplementary figures and images for: Dental Practitioners’ Knowledge and Attitude Towards Ultrasonography: A Cross‐Sectional Study at a South African University
Source: Int J Dent. 2026 Jan 16;2026:7601760. doi: 10.1155/ijod/7601760 (PMC12811406; doi:10.1155/ijod/7601760)

## Supplementary material 1 - Questionnaire


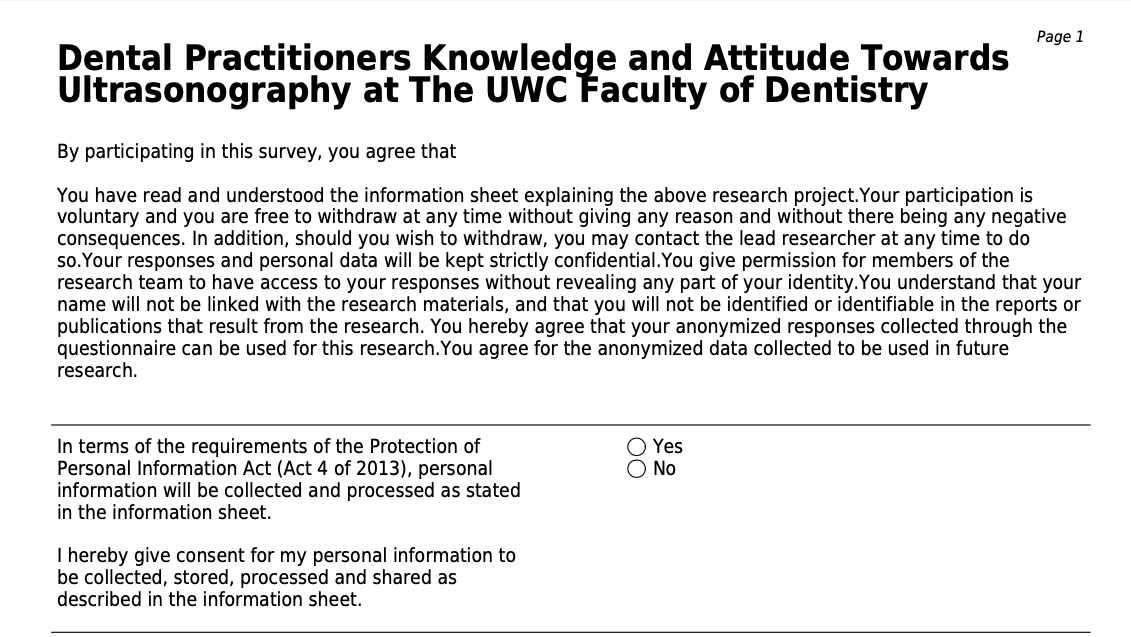

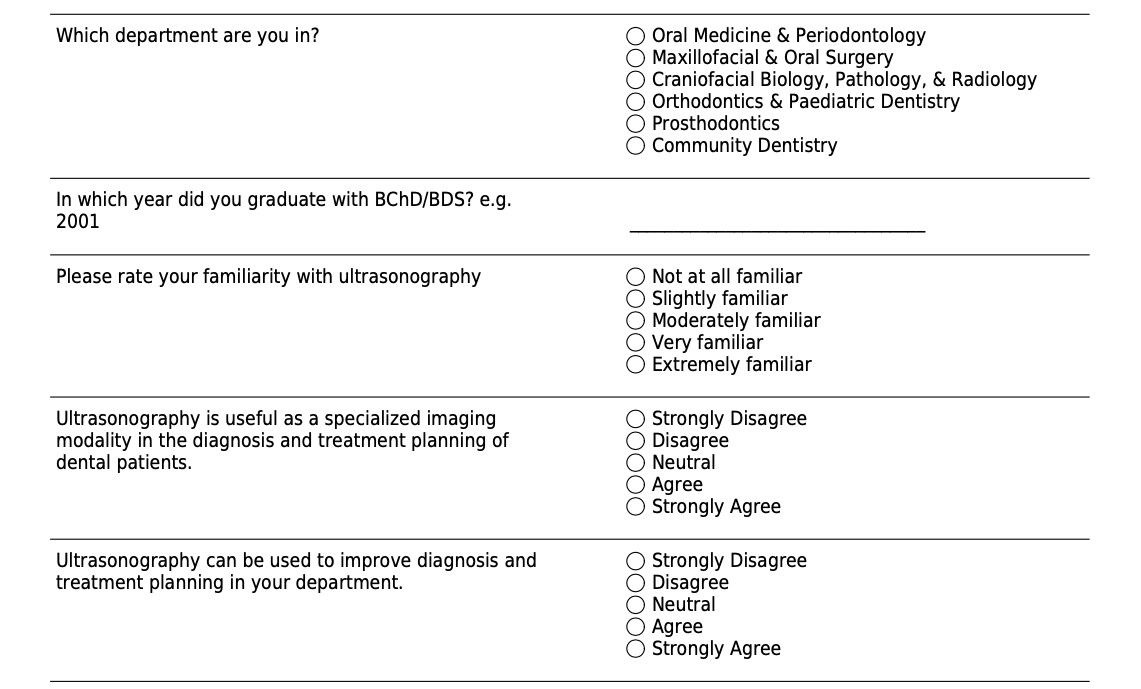

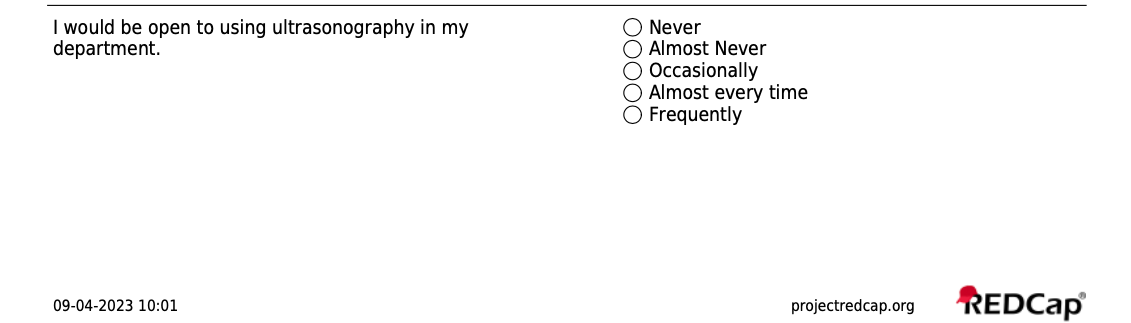

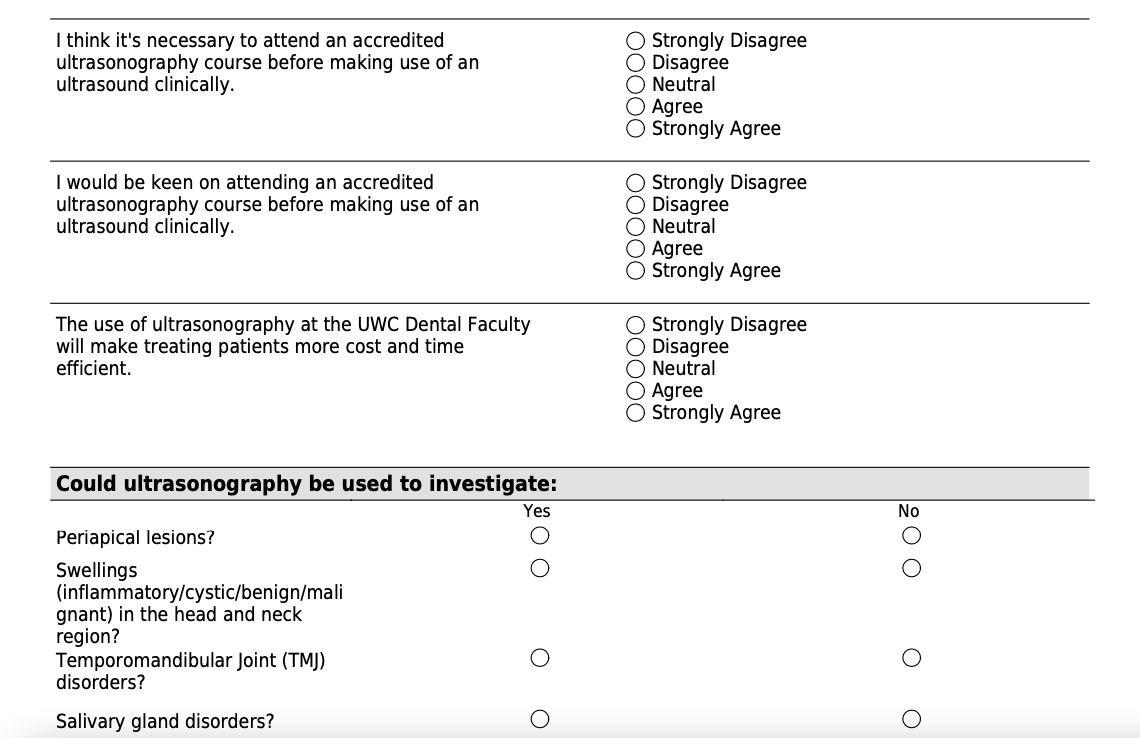

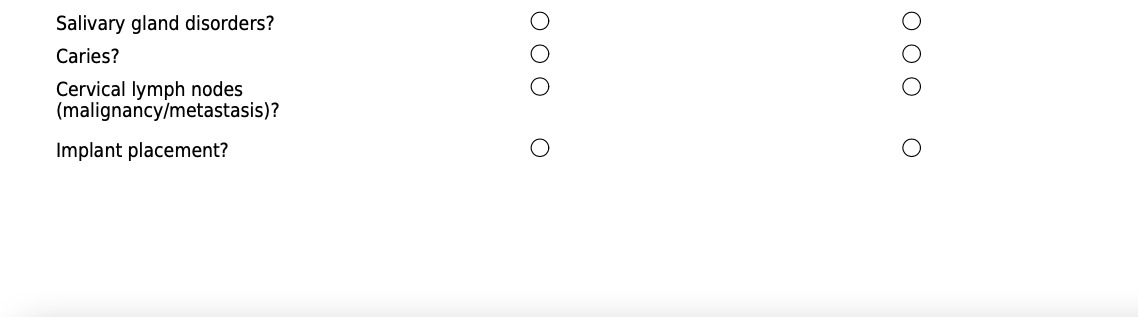

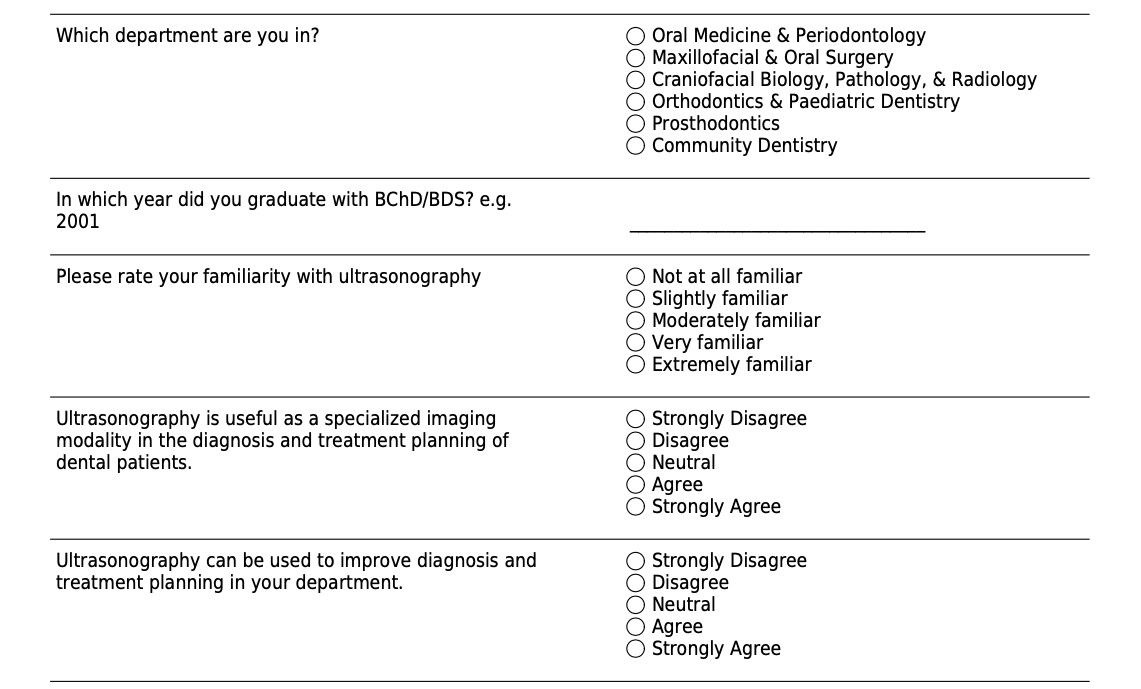

Supplement: Supplementary file 1 — Supporting Information Online questionnaire sent out to all dental practitioners. ∗Referenced on page 5, under “sample size and procedure”. [file IJOD-2026-7601760-s001.docx]
